# Modeling high school timetabling with bitvectors

**DOI:** 10.1007/s10479-016-2220-6

**Published:** 2016-07-22

**Authors:** Emir Demirović, Nysret Musliu

**Affiliations:** Database and Artificial Intelligence Group, Technische Universität Wien, Vienna, Austria

**Keywords:** SMT, High school timetabling, Modeling, Bitvectors, Local search

## Abstract

High school timetabling (HSTT) is a well known and wide spread problem. The problem consists of coordinating resources (e.g. teachers, rooms), times, and events (e.g. lectures) with respect to various constraints. Unfortunately, HSTT is hard to solve and just finding a feasible solution for simple variants of HSTT has been proven to be NP-complete. We propose a new modeling approach for HSTT using bitvectors in which constraint costs of the general HSTT can be calculated using bit operations. This model allows efficient computation of constraint costs making it useful when implementing HSTT algorithms. Additionally, it can be used to solve HSTT with satisfiability modulo theory (SMT) solvers that support bitvectors. We evaluate the performance for our bitvector modeling approach and compare it to the leading engine KHE when developing local search algorithms such as hill climbing and simulated annealing. The experimental results show that our approach is useful for this problem. Furthermore, experimental results using SMT are given on instances from the ITC 2011 benchmark repository.

## Introduction

The problem of high school timetabling (HSTT) is to coordinate resources (e.g. rooms, teachers, students) with times in order to fulfill certain goals (e.g. scheduling lectures). Every high school requires some form of timetabling which is a well known and wide spread problem. The difference between a good and a bad timetable can be significant, as timetables directly contribute to the quality of the educational system, satisfaction of students and staff, etc. Every timetable affects hundreds of students and teachers for prolonged amounts of time, since each timetable is usually used for at least a semester, making HSTT an extremely important and responsible task. However, constructing timetables by hand can be time consuming, very difficult, and error prone. Thus, developing high quality algorithms which would generate automatically timetables is of great importance.

Unfortunately, high school timetabling is hard to solve and just finding a feasible solution of simple variants of High School Timetabling has been proven to be NP-complete (Even et al. 1975). Apart from the fact that problems that need to be solved can be very large and have many different constraints, high school timetabling requirements vary from country to country and because of this many variations of the timetabling problem exist. Nevertheless, a lot of research has been done and HSTT is still an active field of research, even having its own specific HSTT competition ITC 2011.^1^

In order to standardize the formulation for HSTT, researchers have recently proposed a general high school timetabling problem formulation (Post et al. 2014) called XHSTT. This formulation has been endorsed by the Third International Timetabling Competition 2011 (ITC 2011) (Post et al. 2013, 2014) which attracted 17 competitors from across the globe. In this work, we consider the general HSTT problem formulation (XHSTT).

When developing HSTT algorithms, modeling aspects are very important from a practical side, as a good model will allow efficient implementations of HSTT algorithms. However, for a complex problem such as general HSTT, finding good models is a challenging task because of the presence of a large number of different constraints. Therefore, the problem of developing HSTT algorithms is two fold: one must research good algorithmic strategies, while also having efficient data structures or models which will allow fast implementations.

The main contributions of this paper are as follows:

- We present a new modeling of the general HSTT problem (XHSTT) with bitvectors. We model all constraints, except those that deal with resource assignments. With our approach, we can model 23 out of 39 used instances. We considered instances that were used in the International Timetabling Competition 2011 (ITC 2011) and ones which were carefully selected by ITC 2011 after the competition.
- By using this model, we are able to provide an efficient model useful for local search algorithms such as hill climbing and simulated annealing. Additionally, the model is used to encode XHSTT as a Satisfiability Modulo Theory (SMT) problem.
- We give an experimental evaluation of the bitvector approach by comparing it to the leading engine KHE on a simple hill climbing and simulated annealing algorithm. The bitvector approach shows very good results on these algorithms.
- We provide experimental results for the SMT approach using both artificial and real-world instances, all of which were taken from the Third International Timetabling Competition 2011 benchmark repository.

This paper is an extention of the work presented in PATAT 2014 (Demirović and Musliu 2014b). The rest of the paper is organized as follows: in Sect. 2, we present the problem description, followed by related HSTT work in Sect. 3. In the main Sect. 4, we describe the modeling of XHSTT as bitvectors. In Sect. 5, we present computational results. Finally, conclusions are given in Sect. 6.

## Problem description

In our research we consider the general formulation of the High School Timetabling problem (called XHSTT), as described in Post et al. (2014).

High school timetabling has been studied extensively in the past. However, a lot of work has been done in isolation, because different countries have different educational systems and this resulted in many timetabling formulations. It was difficult to compare algorithms and the state-of-the-art was unclear. To solve this issue and encourage timetabling research, researchers have recently agreed on a standardized general timetabling formulation called XHSTT (Post et al. 2014). This formulation was general enough to be able to model different education system from different countries and was endorsed by the International Timetabling Competition 2011. This is the formulation we use in this work.

The general high school timetabling formulation specifies three main entities: times, resources, and events. Times refer to discrete time units which are available, such as Monday 9:00–10:00 and Monday 10:00–11:00. Resources correspond to available rooms, teachers, students, etc. The main entities are the events, which in order to take place require certain times and resources. An event could be a Mathematics lecture, which requires a math teacher (which needs to be determined) and a specific student group (both the teacher and the student group are considered resources) and two units of time (two *times*). Events are to be scheduled into one or more $solution\ events$ or *subevents*. For example, a Mathematics lecture with total duration of four hours can be split into two subevents with duration two, but can be scheduled as one subevent with duration four (constraints may impose further constraints on the durations of subevents).

The aim of HSTT is to find a schedule, by assigning times and resources to events in such a way that all hard constraints are satisfied and that the sum of soft constraints weights is minimized.

Constraints impose limits on what kind of assignments are legal. These may constrain that a teacher can teach no more than five lessons per day, that younger students should attend more demanding subjects (e.g. Mathematics) in the morning, etc. It is important to differentiate between hard constraints and soft constraints. The former are very important constraints which are given precedence over the latter, in the sense that any single violation of a hard constraint is more important than all violations of the soft constraints combined. Thus, one aims to satisfy as many hard constraints as possible, and then optimize for the soft constraints. Each constraint has a nonnegative cost function associated with it, which penalizes assignments that violate it. The goal is to first minimize the hard constraint costs and then minimize the soft constraint costs. In the general formulation, any constraint may be declared hard or soft and no constraint is predefined as such, but rather left as a modeling option based on the concrete timetabling needs. Additionally, each constraint has several parameters, such as to which events or resources it applies, to what extent it applies to (e.g. how many idle times are acceptable during the week), its weight, and other properties, allowing great flexibility.

We now give an informal overview of all the constraints in XHSTT [as given in Post et al. (2014)]. There is a total of 16 constraints (plus preassignments of times or resources to events, which are not listed).

Constraints related to events:

1. Assign time constraints—assign the specified amount of times to specified events.
2. Split events constraints—limits the minimum and maximum durations of subevents and the amount of subevents that may be derived from specified events. Distribute split events (below) gives further control on the subevents.
3. Distribute split events—limits the number and duration of subevents for specified events.
4. Prefer times constraints—when assigning times to events, specified times are preferred over others.
5. Avoid split assignments—for all subevents derived from an event, assign the same resources.
6. Spread events constraints—specified events must be spread out during the week.
7. Link events constraints—specified events must take place simultaneously.
8. Order events constraints—specified events must be scheduled one after the other with a specified number of times in between.

Constraints related to resources:

1. Assign resource constraints—assign specified resources to specified events.
2. Prefer resources constraints—when assigning resources to events, specified resources are preferred over others.
3. Avoid clashes constraints—specified resources cannot be used by two or more subevents at the same time.
4. Avoid unavailable times—specified resources cannot be used at specified times.
5. Limit idle times constraints—specified resources must have their number of idle times lie between given values within specified time groups.
6. Cluster busy times constraints—specified resources’ activities must all take place within a minimum and maximum amount of time groups.
7. Limit busy times constraints—the amount of busy times within specified time groups should lie between given values.
8. Limit workload constraints—specified resources must have their workload lie between given values.

As we describe the modeling, we will give more details on each constraint.

## Related work

For HSTT, both heuristic and complete methods have been proposed. Heuristic methods were historically the dominating approach, as they were able to provide good solutions in reasonable amounts of time even when dealing with large instances, albeit not being able to obtain or prove optimality. Recently complete methods have been proposed and had success in obtaining good results and proving bounds, but require significantly more time (days or weeks).

All of the best algorithms in the International Timetabling Competition 2011 (ITC 2011) were algorithms based on heuristics. The winner was the group GOAL, followed by Lectio and HySST. In GOAL, an initial solution is generated, which is further improved by using simulated annealing and iterated local search, using seven different neighborhoods (Brito et al. 2012). Lectio uses an adaptive large neighborhood search (Sørensen et al. 2012) with nine insertion methods based on the greedy regret heuristics (Sørensen and Stidsen 2012) and fourteen removal methods. HySST uses a hyper-heuristic search (Kheiri et al. 2012).

Afterwards, the winning team of ITC 2011 have developed several new variable neighborhood search (VNS) approaches (Fonseca and Santos 2014). All of the VNS approaches have a common search pattern: from one of the available neighborhoods, a random solution is chosen, after which a descent method is applied and a solution is accepted if it is better than the previous best one. Each iteration starts from the best solution. The most successful of the VSN algorithms was the skewed variable neighborhood in which a relaxed rule is used to accept the new solution, taking into consideration the cost of the new solution as well as its distance from the best solution. A related approach is the late acceptance hill climbing for XHSTT (Fonseca et al. 2015), in which a solution is accepted based on its comparison with the previous *k* solutions, where *k* is a parameter.

Kingston (2014) introduced an efficient heuristic algorithm which directly focuses on repairing *defects* (violations of constraints). Constraint violations are examined individually and specialized procedures are developed for most constraints to repair them. The algorithm is designed to provide high quality solutions in a low amount of time, but does not necessarily outperform other methods with respect to quality of solution.

XHSTT has been modeled with Integer Programming in Kristiansen et al. (2015). This complete approach is able to compute good (and in some cases optimal) solutions as well as lower bounds over longer periods of time using Gurobi (a commercial optimization solver). Additionally, a Large Neighborhood Search with IP has also been developed in Sørensen and Stidsen (2014) which is more efficient than pure IP when given a limited time.

Another complete approach is the maxSAT approach proposed in Demirović and Musliu (2014a). Most of the XHSTT instances could be modeled as a Partial Weighted maxSAT problem and are then solved by a maxSAT solver. The approach can yield good (in some cases optimal) results, although it too requires longer running times.

Additionally, several Integer Programming based techniques have been introduced for similar HSTT problems which are able to provide bounds and good solutions after longer runs (Santos et al. 2012; Sørensen and Dahms 2014; Sørensen and Stidsen 2013), as well as fix-and-optimize IP based hybrid approaches for some instances (Dorneles et al. 2014).

Even though significant work has been done for HSTT, many problems are still not solved efficiently or optimally. Therefore, calculating high quality solutions and providing new modeling approaches are important issues in this domain.

## Modeling XHSTT with bitvectors

In this section we propose a bitvector modeling for XHSTT. The main idea is to provide a simple modeling approach that can be used in different solving techniques. All of the constraint costs are obtained by using bitvector operations. We first introduce basic bitvector definitions and operations used. Then, we define the variables used for modeling XHSTT with bitvectors, followed by the description of XHSTT constraints with bitvectors.

### Basic bitvector definitions

A bitvector is a vector of bits. The size of the vector is arbitrary, but fixed. Standard operations (e.g. *addition*, *and*, *or* operations on bitvectors) and predicates (e.g. equality) are defined over bitvectors and an instance consists of a conjunction of predicates. We use prefix notation, which is common for most SMT solvers, with the addition of brackets and comas in order to ease reading. For example, in infix notation one would write $(a = b)$, while in prefix notation the same expression would be written as $({=}a \ b)$, while we choose to write $({=}(a, b))$.

Most operations are interpreted as usual and all bitvector operands are of the same length. In the following we present some of the notations we use, in which $bv_a$ and $bv_b$ are bitvectors and *k* is a constant integer:

- $$inv(bv_a)$$—inverts $$bv_a$$ bits (e.g. $$inv(1011001) = 0100110$$).
- $add(bv_a, bv_b)$—adds two bitvectors in the same way two unsigned integers would be added (overflow might occur).
- $or(bv_a, bv_b)$—performs bitwise *or* on its operands. When applying *or* to each bitvector $bv_a$ from some set *BV*, we use the following notation: $\bigvee _{bv_a \in BV}(bv_a)$
- $$lshift(bv_a, k)$$—applies noncyclic left shift by *k* operation on $$bv_a$$ (e.g. $$lshift(10011, 2) = 01100 $$).
- $rshift(bv_a, k)$—similar to *lshift*, but uses right shifting.
- $extract(bv_a, k)$—returns the *k-th* bit of $bv_a$

#### Variables

For each event *e* (e.g. a lesson), we create a number of bitvectors all of length *n*, where *n* is the number of times available in the instance. The vectors along with their meanings are as follows:

- $Y_{e}$—the *i-th* bit is set (a bit is *set* if it has value 1) if the event is taking place at time *i* and is not set otherwise. In XHSTT terminology, $Y_{e}$ covers all subevents of event *e*. This implies that two subevents of the same event can never clash in this representation.
- $S_{e}$—the *i-th* bit is set if the *i-th* time is declared as a starting time for event *e* and is not set otherwise.
- $K_{(e, d)}$—the *i-th* bit is set if the *i-th* time is declared as a starting time of duration *d* for event *e* and is not set otherwise.

As an example of the above variables, take the following bitvectors:1From $Y_e$, we see that event *e* (e.g. a Math lesson) is taking place at time 1, 2, 5, and 6, because those bits are set within $Y_e$. Similarly, times 1, 5, and 6 are labeled as starting times from $S_e$, meaning event *e* has been split into three subevents. Time 1 is labeled as a double lesson by $K_{(e,2)}$, while times 5 and 6 as lessons of duration 1 by $K_{(e,1)}$. Note that time 5 could have also been labeled as a double lesson instead of having two lessons of duration 1. Reasons for choosing one possibility over the other is regulated by constraints.

In the formal specification of XHSTT, any time can be defined as a starting time because events can be split into multiple subevents. One could regard a starting point as a time *t* where a lecture takes place, but has not taken place at $t-1$. However, while this is true, this cannot be the only case when a time would be regarded as a starting time, since e.g. time $t = 5$ and $t = 6$ might be interpreted as *last time of Monday* and *first time of Tuesday* and an event could be scheduled at both of these times, but clearly we must regard both times as starting times, since a double lecture does not extend over such long periods of time. Therefore, any time can in general be regarded as a starting time. It is of interest to note that the previous assignment, by the general formulation, could also be treated as a double lesson for the purpose of constraints, even though it extends over two days. Constraints give more control over these kind of assignments.

Note that our model, in order to capture the complete search space for the problem, must account for all possible combinations of the number of subevents for each event. For example, an event of duration 3 can be split into three different ways: one subevent of duration three, two subevents of durations one and two, or three subevents of duration one. Therefore, we cannot assign a bitvector for each subevent in advance because we do not know before hand in how many subevents will a particular event be split into. Due to this we must take into account all possibilities. The equations model all these possible combinations of (nonclashing) subevents.

Formalities that are tied to starting times with regard to the specification are expressed as follows:

If a starting time for event *e* has been assigned at time *t*, then the corresponding event must also take place at that time (the set *E* is the set of all events):

2 $$\begin{aligned} \bigwedge _{e \in E} ({=}(or(S_{e}, Y_{e}), Y_{e})) \end{aligned}$$

When modeling with bitvectors it is common to have formulas of the form $({=}(bv_a, some\ logical\ expression))$, like the one above. This ensures that the bitvector $bv_a$ is equal to the specified logical expression. In Eq. (2), we encode that $Y_{e}$ is equal to $(or(S_e, Y_e))$, meaning that there are no bits set in $S_{e}$ which are not also set in $Y_e$, but it can be that some bits in $Y_e$ are set which are not set in $S_e$. This is the behavior we want to capture, because if some times are declared to be starting times (the bits set in $S_e$), then surely the event in question must take place at those times (hence asserting the bits set in $Y_e$), but since they can last longer than one time it can be the case that $Y_e$ has bits set in position where $S_e$ does not.

Event *e* starts at time *t* if *e* is taking place at time *t* and it is not taking place at time $(t-1)$:

3 $$\begin{aligned} \bigwedge _{e \in E} ({=}(or(and(Y_{e}, lshift (inv(Y_{e}), 1)), S_{e}), S_{e})) \end{aligned}$$

Note that the ordering of the application of *inv* and *lshift* is important. With the application of $exp_1 = (and(Y_{e}, lshift(inv(Y_{e})))$, we will get a bitvector which has its *i-th* bit set iff $Y_e$ has its *i-th* bit set and its $(i-1)$ *-th* bit is not set. Then, similarily to Eq. (2), with the application of ${=}(or(exp_1, S_e), S_e)$ we ensure that $S_e$ has bits set at least in every position as in bitvector $exp_1$, which is what we want to capture: every time we have the situation that a (sub)event is taking place at time *i*, but has not taken place at time $(i-1)$, we declare that time a starting time for said event (note that other times can be starting times too).

If time *t* has been set as a starting time, associate a duration with it (*D*(*e*) is the set of durations that subevent of event *e* can take):

4 $$\begin{aligned} \bigwedge _{e \in E}\left( {=}\left( \left( \bigvee _{d \in D(e)} K_{(e,d)}\right) , S_e\right) \right) \end{aligned}$$

By setting a bit in position *i* in $S_e$ we ensure that at least one $K_{(e,d)}$ will have an *i-th* bit set. Later on through Eq. (8) we ensure that exactly one $K_{(e,d)}$ will have such bit set.

If a subevent of event *e* of duration *d* has been assigned a starting time at time *t* and event *e* is also taking place at time $t+d$, then assign time $t+d$ as a starting time (*D*(*e*) is the set of possible durations subevents of *e* might take):

5 $$\begin{aligned} \bigwedge _{\begin{array}{c} e \in E \\ {d \in D(e)} \end{array}}({=}(or(and(lshift(K_{(e,d)}, d), Y_{e}), S_{e}), S_{e}) ) \end{aligned}$$

The formula $exp = and(lshift(K_{(e,d)}, d), Y_{e})$ will result in a bitvector which has its *i-th* bit set if event *e* is taking place at time *i* and event *e* has been declared to have a starting point at *i–d* time of duration *d*. In other words, event *e* started at *i–d* and was declared to last *d* times, but after *d* times event *e* is still taking place. Therefore, we want to ensure that event *e* will also have a starting point at time *i*. This is then done in a similar fashion to before: $({=}(or(exp, S_e), S_e))$.

When a bit in $K_{(e,d)}$ is set, ensure that the event in question must take place for *d* consecutive times during this specified time. In order to do this, we define a helper bitvector $Y_{e}^d$ which will have its *i-th* bit set if starting from time *i* event *e* has *d* consecutive bits set. For example, if $Y_e = (0, 0, 1, 1, 1, 0)$, then $Y_{e}^3 = (0, 0, 0, 0, 1, 0)$ and $Y_{e}^2 = (0, 0, 0, 1, 1, 0)$ (recall that the right most bit represents time 0). Bitvector $Y_{e}^d$ can be computed by taking the *and* of all of $rshift(Y_{e}, k)$ for $k = 0{\ldots }(d-1)$ (with $rshift(Y_e, 0) = Y_e$). We now proceed with the constraint encoding:

6 $$\begin{aligned} \bigwedge _{\begin{array}{c} e \in E \\ {d \in D(e)} \end{array}}\left( {=}\left( or\left( K_{(e,d)}, and\left( Y_{e}^d, K_{(e,d)}\right) \right) , K_{(e,d)}\right) \right) \end{aligned}$$

The expression $exp = and(Y_{e}^d, K_{(e,d)})$ is a bitvector which has its *i-th* bit set if event *e* has *d* consecutive bits set starting from time *i* and has a starting time of duration *d* at time *i*. In order to ensure that when a bit in $K_{(e,d)}$ is set there must be *d* consecutive bits set in $Y_e$ starting from time *i*, we encode: $({=}(or(exp, K_{(e,d)}),K_{(e,d)}))$.

If an event *e* has a subevent of duration *d* starting at time *i* (the *i-th* bit set in $K_{(e,d)}$), make sure that no other starting time can be set within the duration of that subevent. In order to do this, we define a helper bitvector $K_{(e,d)}^k$ as:

7 $$\begin{aligned} \bigwedge _{\begin{array}{c} e \in E \\ d \in D \end{array}}\left( {=}\left( \bigwedge _{i=0{\ldots }k}(inv(rshift(K_{(e,d)}, i))), K_{(e,d)}^{k}\right) \right) \end{aligned}$$

Bitvector $K_{(e,d)}^k$ will have its *i-th* bit set if there is no bit set at time *i* nor in any of the next *k* times in $K_{(e,d)}$. We use this helper bitvector to encode the constraint:

8 $$\begin{aligned} \bigwedge _{\begin{array}{c} e \in E \\ d_1 \in D \end{array}} \left( {=}\left( and\left( K_{(e,d_1)}, \bigwedge _{\begin{array}{c} d_2 \in D \\ d_1 \ne d_2 \end{array}}\left( K_{(e,d_2)}^{(d_1-1)}\right) \right) , K_{(e,d_1)}\right) \right) \end{aligned}$$

With $exp = \bigwedge _{d_2 \in D}(K_{(e,d_2)}^{d_1-1})$ we compute a bitvector which has its *i-th* bitvector set if there is no bit set at time *i* in any of the $K_{(e,d_2)}$ (with $d_1 \ne d_2$) nor in any of the next $d-1$ times. Therefore, only in $K_{(e, d_1)}$ can bits in these times be set. Then, $({=}(K_{(e,d_1)}, and(K_{(e, d_1)}, exp))$ ensures that if $K_{(e, d_1)}$ has a bit set at time *i*, it must be the case that no other $K_{(e,d_2)}$ (with $d_1 \ne d_2$) has its bit at *i* nor in the next $k-1$ times.

With this constraint, we conclude constraints regarding starting time definitions. We now proceed with cardinality constraint encodings followed by high school timetabling constraint encodings.

### Cardinality constraint encodings

An important constraint that arises often is to determine the number of set bits in a bitvector, as well as to impose penalties if the appropriate number of bits are not set. E.g. if an event must take place for two hours, then exactly two bits in its $Y_e$ must be set.

Let us define a unary operation $reduceBit(bv_a) = bv_a \wedge sub(bv_a, 1)$. When applied to $bv_a$, as the name suggests, it produces a new bitvector which has one less bit set then $bv_a$ (for the special case $bv_a = 0$, it returns 0). For example:9The original bitvector had three bits set, while the produced one has two bits set. The *reduceBit* operations is an important part for defining cardinality constraints.

In order to ensure that $at\ least\ k$ bits are set in a bitvector, we apply *reduceBit* $k-1$ times and require that the resulting bitvector must be different from zero. For $at\ most\ k$, we apply *reduceBit* *k* times and constrain that the resulting bitvector must be equal to zero. For $exactly\ k$ we encode $at\ least\ k$ and $at\ most\ k$. For example, asserting that $at\ least\ 3$ bits are set is done in the following way:10Since the final bitvector, which we have obtained by applying reduceBit twice, is different from the zero bitvector we conclude that $at\ least\ 3$ bits are set in $bv_a$.

It is important to note that when using the modeling for local search, bitvectors can be implemented using binary integers and standard binary operations over bits can be used. Additionally, most modern processors have special operations for determining the number of bits set in an integer. These operations are called *population counts* or *hamming weight instructions*. We recommend using them if possible as they are more efficient than repeatedly applying the defined reduce operation when implementing local search algorithms with bitvectors.

#### Soft cardinality constraints

A similar technique to the one previously described is used for soft cardinality constraints. For $at\ least\ k$, it is asserted before each application of *reduceBit* and after the last application of *reduceBit* that the current bitvector is different from zero and is penalized by some weight if it is not the case. For example, asserting that $at\ least\ 2$ bits are set is done in the following way for the soft version:11Note that we checked for penalties in two cases (for the initial bitvector $bv_a$ and $reduceBit(bv_a)$), but only one case was penalized in this particular case.

For $at\ most\ k$, a similar algorithm is used: *reduceBit* is applied *k* times as in the regular cardinality constraint version and then *bitReduce* is applied $n-k$ times to this bitvector (*n* is the size of $bv_a$) and before each application it is asserted that the current bitvector is zero and is penalized by some weight if it is not the case. Note that if we have some hard constraint limiting the maximum number of bits that may be set in a bitvector to some $k_{max}$, we do not perform the second part of the algorithm $n-k$ times, but rather just $k_{max} - k$ times. This is used frequently while modeling for SMT.

The penalty weights depend on the cost function chosen and this is discussed in the next section.

#### Cost functions

The way the penalty weights are assigned depends on the constraint that is being modeled. Following XHSTT, we use three different penalty schemes: Linear, Quadratic, and Step. The Linear scheme penalizes linearly to its violation, the Quadratic scheme penalizes by squaring it, and the Step scheme assigns a penalty of one if there is a violation (regardless of how severe) and zero otherwise. These values are then multiplied by a weight *w* which is given in the constraint that is being modeled.

For the example used in Eq. (11), the linear scheme assigns a penalty of *w* to each violation, the quadratic one would assign *w* to the first and $3*w$ to the second, while step would assign *w* and 0.

### Constraints

Each constraint has a set of points of application and each point generates a deviation. The cost of the constraint is obtained by applying a cost function on each deviations, multiplying it by a weight, and then summing up all these values. There are three different cost functions, as discussed in Sect. 4.2.2.

When modeling XHSTT as SMT, we simplify the objective function by not tracking the infeasibility value, rather regarding it as zero or nonzero. By doing so we simplify the computation for the SMT solver, possibly offering faster execution times. However, when using the bitvector modelings for implementing local search algorithms, both hard and soft costs are tracked.

*E*, *T* and *R* are sets of events, times and resources, respectively. Each constraint is applied to some subset of those three, denoted by $E_{spec}$, $T_{spec}$ and $R_{spec}$. These subsets are naturally in general different from constraint to constraint. Note that it is possible to have several constraints of the same type, but with different subsets defined for them.

We present encodings used in the experimental results, in which we assume that all resources are already assigned to events. We make this assumption as this eases the modeling and readability of the constraints. Later on we provide a description on how this limitation can be overcome.

Unless explicitly stated, soft constraints are implemented by using soft instead of hard cardinality constraints for the key equations which encode the limitations enforced by the constraint. In cases when this differs, we provide an explanation.

#### Assign time constraints

Every event must be assigned a given amount of time. For example, if a lecture lasts for two hours, two times must be assigned to it.

Each event’s $Y_{e}$ vector must have exactly *d* bits set, where *d* is the duration of the event:

12 $$\begin{aligned} \bigwedge _{e \in E_{spec}} (exactly\_d[ Y_{e} ] ) \end{aligned}$$

If the constraint is specified as soft, then instead of the equation above we would use the soft cardinality encoding for $atLeast\_d$ and a hard cardinality constraint $atMost\_d$ with $Y_e$. Points of applications are events and the deviation for each event is calculated as the number of times not assigned to the event.

#### Split events constraints

This constraint has two parts. The first part limits the number of starting times an event may have in the solution. The second part limits the duration of the event for a single subevent.

For example, if four times must be assigned to a Mathematics lecture, we may limit that the minimum and maximum duration of a subevent is equal to 2, thus ensuring that the lecture will take place as two blocks of two hours, forbidding having the lecture performed as one block of four hours.

This constraint specifies the minimum $A_{min}$ and maximum $A_{max}$ amount of starting times for the specified events:

13 $$\begin{aligned} \bigwedge _{e \in E_{spec}} (atLeast\_A_{min}[ S_{e} ] \wedge atMost\_A_{max}[ S_{e} ] ) \end{aligned}$$

In addition, this constraint also imposes the minimum $d_{min}$ and maximum $d_{max}$ duration for each subevent:

14 $$\begin{aligned} \bigwedge _{\begin{array}{c} e \in E_{spec} \\ {d \in \{i | i < d_{min} \vee i > d_{max}\}} \end{array}}(atMost\_0[K_{(e,d)}] ) \end{aligned}$$

#### Distribute split events constraint

This constraint specifies the minimum $d_{min}$ and maximum $d_{max}$ number of starting times of a specified duration *d*. For example, if $duration(e)=10$, we may impose that the lecture should be split so that at least two starting times must have duration three. The constraint is encoded as follows:

15 $$\begin{aligned} \bigwedge _{e \in E_{spec}}(atLeast\_d_{min}[K_{(e,d)}] \wedge atMost\_d_{max}[K_{(e,d)}]) \end{aligned}$$

#### Prefer times constraints

This constraint specifies that certain events should begin at certain times. If an optional parameter *d* is given, then this constraint only applies to subevents with duration *d*. For example, a lesson of *duration 2* must be scheduled on Monday, excluding the last time on Monday.

Let $P_{e}$ be the bitvector in which the *i-th* bit is set iff *i* is a preferred time. We then encode:

16 $$\begin{aligned} \bigwedge _{e \in E_{spec}} (atMost\_0[ and(\star , inv(P_{e})) ] ) \end{aligned}$$

where $\star$ is either $S_{e}$ or $K_{(e,d)}$, depending on whether the optional parameter *d* is given.

If the constraint is required to be soft and the optional parameter *d* is not given, then the following formula is used instead ($D_e$ is the set of duration event *e* can be subdivided into):

17 $$\begin{aligned} \bigwedge _{e \in E_{spec}} \bigwedge _{k \in D_{e}} (k * atMost\_0[ and( K_{(e,k)}, inv(P_{e})) ] ) \end{aligned}$$

If the optional parameter *d* is given, then instead of $D_e$ we would use the singleton $\{d\}$. The *k* in front of $atMost\_0$ represents that when calculating the weights for violating the constraint, one must consider the deviation *k* times larger than normally (the constraint penalizes misplaced (sub)events of longer duration more).

#### Spread events constraints

Certain events must be spread across the timetable, e.g. in order to avoid situations in which an event would completely be scheduled only in one day.

An event group *eg* is a set of events. Depending on these events, we propose two encodings for this constraint. The first encoding is simpler, but requires that the events in the specified event group cannot share any times. Formally, we require that:

18 $$\begin{aligned} \bigwedge _{eg \in EG_{spec} } \bigwedge _{\begin{array}{c} (e_i, e_j) \in eg^2 \\ e_i \ne e_j \end{array}}\left( {=}\left( and\left( Y_{e_i}, Y_{e_j}\right) , 0\right) \right) \end{aligned}$$

The previous equation holds in all of the instances considered in this paper because events in the event groups share a common resource and avoid clash constraints prevents them from having shared times. Therefore, we use the the simpler encoding for modeling. We now proceed with this description and give the general case afterwards.

Let $Z_{eg}$ be a bitvector which has its *i-th* bit set iff an event $e \in eg$ has a starting time at time *i*. This is obtained by applying *or* to all of the appropriate $S_{e}$ vectors.

This constraint specifies event groups to which it applies, as well as a number of time groups (sets of times) and for each such time group the minimum and maximum number of starting times events from a given event group must have within times of that time group. Let $TG_{spec}$ denote this set of sets of times and let $mask_{tg}$ be the bitvector which has its *i-th* bit set iff *i* is a time of time group *tg*. We define helper bitvectors $C_{(tg, eg)}$:

19 $$\begin{aligned} \bigwedge _{\begin{array}{c} tg_i \in TG_{spec} \\ eg \in EG_{spec} \end{array}} \left( {=}\left( C_{(tg, eg)}, and\left( Z_{eg}, mask_{tg}\right) \right) \right) \end{aligned}$$

This constraint specifies the minimum $d_{i}^{min}$ and maximum $d_{i}^{max}$ amount of starting times within a given time group $tg_i$:

20 $$\begin{aligned} \bigwedge _{\begin{array}{c} tg_i \in TG_{spec} \\ eg \in EG_{spec} \end{array}} \left( atLeast\_d_i^{min} [C_{(tg_i, eg)}] \wedge atMost\_d_i^{max} [C_{(tg_i, eg)}]\right) \end{aligned}$$

If this constraint is used as a soft constraint, the soft cardinality constraint is used instead. Points of application are event groups (not events) and deviations are calculated as the number of set bits by which $C_{(tg_i, eg)}$ falls short of the minimum or exceeds the maximum.

As discussed previously, the provided encoding holds only if Eq. (18) holds. Otherwise, the encoding given above will not be correct, because $Z_{eg}$ does not account for more than one starting time at any time. Therefore, for each time *t* we would need to count how many starting times (from the events in the event group) take place at that time *t*. This can be done by using a helper bitvector $Q_{(tg, eg)}$ defined as:

21 $$\begin{aligned} \bigwedge _{\begin{array}{c} tg_i \in TG_{spec} \\ eg \in EG_{spec} \end{array}} \left( {=}\left( Q_{(tg_i, eg)}, \bigvee _{e_j \in eg}\left( lshift\left( extendBV(and(S_{e}, mask_{tg_i}), |T|*j), |T|*j\right) \right) \right) \right) \end{aligned}$$

Here the indicies *i* and *j* represent the position of a time group or event within its time group ($i=0{\ldots }(|tg_i|-1)$ or event group $j=0{\ldots }(|eg|-1)$). The function $extendBV(bv_a, n)$ extends the bitvector $bv_a$ to the size of *n* by adding the appropriate number of zeros to the end of $bv_a$. We use this function because otherwise *lshift* would remove all information about the starting times due to the length of $S_e$ (which is equal to |*T*|). The resulting bitvector $Q_{(tg, eg)}$ is of size $|eg| * |T|$ (number of events in *eg* multiplied by the number of times in *tg*). The inner *and* operation ensures that only bits related to $tg_i$ are taken into consideration and the *lshift* operations places the bits related to *tg* of the events from *eg* one after the other in $Q_{(tg, eg)}$. We can now encode the constraint:

22 $$\begin{aligned} \bigwedge _{\begin{array}{c} tg_i \in TG_{spec} \\ eg \in EG_{spec} \end{array}} \left( atLeast\_d_i^{min} [Q_{(tg_i, eg)}] \wedge atMost\_d_i^{max} [Q_{(tg_i, eg)}]\right) \end{aligned}$$

#### Link events constraints

Certain events must be held at the same time. For example, physical education lessons for all classes of the same year must be held together. This constraint specifies a certain number of event groups and imposes that all events within an event group must be held simultaneously. Let $EG_{spec}$ denote this set of sets of events and $Z_{eg}$ be a bitvector which has its *i-th* bit set iff an event $e \in eg$ is taking place at time *i*.

We define a helper bitvector $L_{eg}$ whose *i-th* bit is set iff at time *i* at least one event is taking place but not all the events of the specified event group:

23 $$\begin{aligned} \bigwedge _{eg \in EG_{spec}} \left( {=}\left( L_{eg}, \bigvee _{e_i \in eg} and\left( Z_{eg}, inv\left( Y_{e_i}\right) \right) \right) \right) \end{aligned}$$

The constraint is now encoded as:

24 $$\begin{aligned} \bigwedge _{eg \in EG_{spec}}\left( atMost\_0[L_{eg}]\right) \end{aligned}$$

#### Order events constraints

This constraint specifies pairs of events and constrains that there must be a certain number of times in between the last time of the first event and the first time of the second event. Parameters $B_{min}$ and $B_{max}$ are given which define the minimum and maximum time separations between two events and are by default set to zero and the number of times, respectively. The constraint specifies a set of pairs of events to which it applies.

In order to encode this constraint, we define helper bitvectors with the aim of tracking the distance between the two events. The first type of helper bitvectors we define are $MAX_{e}$ and $MIN_{e}$, which have its *i* *-th* bit set iff event *e* is taking place at time *i* but not in any time after or before *i*, respectively:

25 $$\begin{aligned} \bigwedge _{e \in E_{spec}} ({=}(MAX_{e}, and\,(Y_{e}, inv(G_{(e, T)})) \wedge ({=}(MIN_{e}, and(Y_{e}, inv(H_{(e, T)})) ) \end{aligned}$$

Both $MAX_e$ and $MIN_e$ have exactly one bit set. In the above equation *T* is the set of all times, and $G_{(e, T)}$ and $H_{(e, T)}$ are as defined in Sect. 4.3.10 but with $Y_e$ being used instead of $X_{r}$.

The next helper bitvector is $MAX_{e}^{\prime }$, which has the same bit set as $MAX_e$ but also all bits to the right of it. Similar for $MIN_{e}^{\prime }$ except all bits from the left are set. For example, if $MAX_{e_i} = (0, 0, 0, 1, 0)$ and $MIN_{e_j} = (0, 1, 0, 0, 0)$ then $MAX_{e_i}^{\prime } = (0, 0, 0, 1, 1)$ and $MIN_{e_j}^{\prime } = (1, 1, 0, 0, 0)$. This is done for $MAX_e^{\prime }$ by taking the *or* of all bitvectors $rshift(MAX_{e}, i)$ with $i = 0{\ldots }(|T|-1)$. For $MIN_e^{\prime }$, $lshift(MIN_{e}, i)$ is used instead.

We now define a helper bitvector for a pair of events $SEP_{(e_i, e_j)}$, which has its $i-th$ bit set iff time *i* is between the last time of $e_{i}$ and the first time of $e_j$:

26 $$\begin{aligned} \bigwedge _{(e_i, e_j) \in E_{spec}^2} ({=}(inv(or(MIN_{e_j}^{\prime }, MAX_{e_i}^{\prime })), SEP_{(e_i, e_j)})) \end{aligned}$$

Since $MAX_{e_i}^{\prime }$ has all bits set until the last time of $e_i$, and $MIN_{e_j}^{\prime }$ has all bits set after the first time of $e_j$, by taking the *or* of these two vectors we would get a new bitvector which has zeros only in position which are in between the last time of $e_i$ and first time of $e_j$. Therefore, performing an inverse of this would get us the desired bitvector $S_{(e_i, e_j)}$. Note that the order in the pair is important $(e_i, e_j)$: $SEP_{(e_i, e_j)}$ and $SEP_{(e_j, e_i)}$ are two different bitvectors (at least one of the two will be a zero bitvector).

The above statements for $SEP_{(e_i, e_j)}$ hold only if the last time of $e_i$ is before the first time of $e_j$. Therefore, the constraint is encoded as follows, given the specified minimum $d_{min}$ and maximum $d_{max}$ times in between events:

27 $$\begin{aligned}&\displaystyle \bigwedge _{(e_i, e_j) \in E_{spec}^2}(atLeast\_d_{min}[SEP_{(e_i, e_j)}]) \wedge (atMost\_d_{max}[SEP_{(e_i, e_j)}]) \end{aligned}$$

28 $$\begin{aligned}&\displaystyle \bigwedge _{(e_i, e_j) \in E_{spec}^2}(<(MAX_{e_i}, MIN_{e_j})) \end{aligned}$$

If the constraint is specified as a soft constraint, additional modifications and equations are required. We do not discuss the encoding in detail and briefly sketch it instead. The main idea is to consider three cases: when the last time of $e_i$ is before the first time of $e_j$, when the last time of $e_i$ is exactly first time of $e_j$, and when the last time of $e_i$ is after the first time of $e_j$. For each of these cases, we would encode constraints which penalize the objective function only if the given case is satisfied. In order to determine each case, equations similar to Eq. (28) would be encoded, but with <, $=$, and > operators. The penalty equations for the first case would correspond to the same as Eq. (27) but with soft cardinality encodings, for the second case a fixed penalty would suffice, while for the third case an equation similar to Eq. (27) with $SEP_{(e_j, e_i)}$ and soft cardinality encodings would be used.

#### Avoid unavailable times constraints

Specified resources are unavailable at certain times. For example, a teacher might be unable to work on Friday.

Let *UAT* be the bitvector which has its *i-th* bit set if *i* is unavailable time. We encode the constraint as follows:

29 $$\begin{aligned} \bigwedge _{\begin{array}{c} r \in R_{spec} \\ e \in E(r) \end{array}} (atMost\_0[and(Y_{e}, \textit{UAT} )]) \end{aligned}$$

#### Avoid clashes constraints

Specified resources can only be used at most by one event at a time. For example, a student may attend at most one lecture at any given time.

Let *E*(*r*) be the set of events which require resource *r*. For each resource *r*, each time *i* and each combination of two $Y_{e}$ vectors of events from *E*(*r*) at most one bit at the *i-th* location may be set:

30 $$\begin{aligned} \bigwedge _{\begin{array}{c} r \in R \\ e_1, e_2 \in E(r) \\ e_1 \ne e_2 \end{array}} ({=}(and(Y_{e_1}, Y_{e_2}), 0) ) \end{aligned}$$

If the constraint is specified as a soft constraint, a different encoding should be used. Points of application are resources and deviations are calculated as follows: for each time in which the resource is used by two or more events, compute the number of events which require the resource minus one. Then, the sum of all these numbers is the deviation for a single resource.

We give equations which can be used if the cost function is linear, which we have used in our local search bitvector implementation. To do so, first we recursively define auxiliary variables $f_{(r, i)}$ (the index *i* goes from zero):

31 $$\begin{aligned}&\displaystyle \bigwedge _{r \in R } ({=}(0, f_{(r, -1)}) ) \end{aligned}$$

32 $$\begin{aligned}&\displaystyle \bigwedge _{\begin{array}{c} r \in R \\ {e_i \in E(r)} \end{array}} ({=}(or(Y_{e_i}, f_{(r,i-1)}) ), f_{(r, i)}) ) \end{aligned}$$

The constraint cost for the linear case is then encoded as:

33 $$\begin{aligned} \bigwedge _{\begin{array}{c} r \in R \\ {e_i \in E(r)} \end{array}} ( atMost\_0[and(f_{(r, i-1)}, Y_{e_i})] ) \end{aligned}$$

#### Limit idle times constraints

This constraint specifies the minimum and maximum number of times in which a resource can be idle during the times in specified time groups. For example, a typical constraint is to impose that teachers must not have any idle times.

A time *t* is idle with respect to time group *tg* (set of times) iff it is not busy at time *t*, but is busy at an earlier time and at a later time of the time group *tg*. For example, if a teacher teaches classes Wednesdays at *Wed*2 and *Wed*5, he or she is idle at *Wed*3 and *Wed*4, but is not idle at *Wed*1 and *Wed*6. This constraint places limits on the number of idle times for each resource.

To ease the encoding of this constraint, we define a helper bitvector $X_r$ for each resource, such that its *i-th* bit is set if resource *r* is busy at the *i-th* time:

34 $$\begin{aligned} \bigwedge _{r \in R} \left( {=}\left( X_r, \bigvee _{e \in E(r)}(Y_e) \right) \right) \end{aligned}$$

We define two other helper bitvectors: $G_{(r, tg)}$ and $H_{(r, tg)}$. For $G_{(r, tg)}$, the *i-th* bit is set if resource *r* is busy at some time within time group *tg* that takes place after *i*. For $H_{(r, tg)}$, it is similar except it considers times happening before *i*. For $G_{(r, tg)}$, these can be computed by taking *or* of bitvectors $rshift(and(X_{r}, mask_{tg}), k)$ where $k=1{\ldots }n$ and *n* is the number of times in time group *tg*. For $H_{(e, tg)}$ it is similar, except using *lshift* instead of *rshift*. Before finalizing the encoding for this constraint, we define another auxiliary variable.

35 $$\begin{aligned} \bigwedge _{\begin{array}{c} r \in R_{spec} \\ tg \in TG_{spec} \end{array}} ({=}(W_{(r, tg)}, and(inv(X_{r}), and(H_{(r, tg)}, G_{(r, tg)}) ) )) \end{aligned}$$

If for a resource *r* the *i-th* bit in $G_{r, tg}$ and $H_{r, tg}$ is set but not in $X_r$, then the *i-th* bit in $W_{(r, tg)}$ will be set indicating an idle time. We now encode the constraint.

36 $$\begin{aligned} \bigwedge _{r \in R_{spec}}\left( atMost\_idle_{max}\left[ \bigvee _{tg \in TG_{spec}}(W_{(r, tg)})\right] \right) \end{aligned}$$

A similar encoding to the one above is also used, but with $atLeast\_idle_{min}$.

#### Cluster busy times constraints

This constraint specifies the minimum and maximum number of specified time groups in which a specified resource can be busy. For example, we may specify that a teacher must fulfill all of his or her duties in at most three days of the week.

We define a helper bitvector $B_{r}$ for each resource, in which the *i-th* bit is set iff the resource is busy at the *i-th* time group. Let us denote with $tg_i$ the *i-th* time group, and with $B_r(i)$ and $X_r(i)$ the *i-th* bits of $B_r$ and $X_r$,^2^ respectively. We can then encode this constraint as follows:

37 $$\begin{aligned} \bigwedge _{tg_i \in TG} \left( {=}\left( B_{r}(i), \bigvee _{t \in tg_{i}} X_r(t)\right) \right) \end{aligned}$$

This constraint specifies the minimum $b_{tg}^{min}$ and maximum $b_{tg}^{max}$ busy time groups:

38 $$\begin{aligned} \bigwedge _{r \in R_{spec}}(atLeast\_b_{tg}^{min}[B_{r}]) \wedge (atMost\_b_{tg}^{max}[B_{r}]) \end{aligned}$$

#### Limit busy times constraints

This constraint imposes limits on the number of times a resource can become busy within certain a time group, if the resource is busy at all during that time group. For example, if a teacher teaches on Monday, he or she must teach at least for three hours. This is useful in preventing situations in which teachers or students would need to come to school only to have a lesson or two.

A resource is busy at a time group *tg* iff it is busy in at least one of the times of the *tg*. We create a helper bitvector $X_r$ which represents a bitvector which has its *i-th* bit set if resource *r* is busy at time *i*. This can be done by taking the *or* of $Y_e$ for all events which require resource *r*. With $TG_{spec}$ we denote the set of sets of times given by the constraint and encode the constraint as follows:

39 $$\begin{aligned} \bigwedge _{\begin{array}{c} r \in R_{spec} \\ tg \in TG_{spec} \end{array}} (or(atLeast\_b_{min}[and(X_{r}, mask_{tg})], ({=}(and(X_{r}, mask_{tg})), 0) )) \end{aligned}$$

The formula $exp = ({=}(and(X_{r}, mask_{tg})), 0)$ will return *true* if resource *r* is *not* busy within time group *tg*. Therefore, in this case the constraint given above will be satisfied. Otherwise, we force the *atLeast* constraint to be satisfied, limiting the minimum number of times *r* must be busy during that time group. With this, we capture the behavior we would like: if the resource is not busy during the day do not make any further constraints, but if it is busy make sure the resource works for at least $b_{min}$ times. A similar encoding to the one above is also used, but with $atMost\_b_{max}$. Note that in this case *or* represents $logical\ or$, rather than $bitvector\ or$.

If this constraint is used as a soft constraint, the soft cardinality constraint is used instead, although special care must be given as this is a conditional cardinality constraint: if the calculated vector is different from zero then the cardinality constraints need to be fulfilled. Points of application are resources and for each resource its deviation is calculated as the sum of number by which the events group falls short of the minimum or exceeds the maximum for each time group.

#### Extending the model

As mentioned in the beginning, we made the assumption that all resources have been assigned to events, as it is easier to model, implement, and present the formulation. This is a reasonable assumption, as most instances are of this form. Still, a significant part of the instances require assignments of resource to events. Our model can be extended with these requirements by introducing new bitvectors: for each event *e* and resource *r*, a bitvector is created in which the *i-th* bit is set iff resource *r* has been assigned to event *e* at time *i*. With these bitvectors, the other resource assigning constraints (we direct interested readers to Post et al. (2014)) can be encoded in a similar fashion as the ones already presented, along with certain modifications that need to be made to avoid clash constraints. In the general case, this would lead to a significant increase in bitvectors and in turn might lead to longer solutions times, which is why particular cases rather than general ones should be considered (see next paragraph).

Special care needs to be given when doing so with concrete instances, as requirements for resource assignments can be diverse. For example, in instance *SpainInstance* given in the ITC repository, assignments consist of assigning one gym room out of two available. For instance *EnglandStPaul*, rooms need to be assigned and many symmetries appear because all rooms are identical. Hence, it might be a better idea to restrict the number of events at each time to the number of rooms, rather than assigning rooms directly to events. A similar situation arises in *FinlandArtificialSchool*, where there are many rooms, but only three different types and a counting strategy like the one described for *EnglandStPaul* would be more appropriate.

In addition, it may be of interest to simplify the $K_{(e,d)}$ and $S_{e}$ encodings. The general formulation allows a variety of situations to be encoded, but in most instances times are partitioned into days, events do not span over more than one day, and an event has at most one starting time per day. With this in mind, we could simplify the encoding of $K_{(e, d)}$ and $S_{e}$ from Sect. 4.1.1. One way to do so would be to forbid the appropriate $K_{(e,t)}$ variables so that events cannot span over multiple days and simply state that if an event has *n* consecutive times followed by an unset bit in a day that it has a starting time with duration *n* (the corner case being when the event ends at the last time of the day). This would lead to simpler encodings which would be potentially easier to solve than the general formulation.

When using the described model for implementing local search algorithms, one must decide whether to allow situations in which an event may clash with itself. For example, we may split a Mathematics lesson of four hours into two lessons of two hours. When scheduling this event, we schedule the first and second subevent to take place on the first or second time. However, since both subevents are of length two, the event will clash with itself. If such a situation is considered legal, then certain modifications to the present modeling need to be taken care of, as individual subevents need to be tracked and used in some constraints. For example, if an event is self clashing, when calculating its spread events constraints one must check each of its subevents rather than using $Y_{e}$, since it may be the case that two subevents are scheduled to take place at the same time. In our local search implementation, we allowed self clashing events, since the KHE engine and state-of-the-art algorithms for XHSTT define this as a legal solution, although we note that forbidding self clashes significantly simplifies the implementation.

We note that our model cannot be directly used by constructive local search algorithms which would start from a solution with no assignments and construct a solution according to some heuristic. The reason is that when calculating the deviations for each constraint, it is assumed that all events are assigned the appropriate amount of times by Assign Times Constraint. Therefore, if one wishes to use our model with such an algorithm this needs to be taken into consideration and appropriate modifications should be performed when calculating deviations for constraints which are affected.

## Computational results

In this section we evaluated our bitvector model by using simple implementations of local search algorithms such as hill climbing and simulated annealing, as well as solving XHSTT with SMT. All tests were performed on (Intel Core i3-2120 CPU @ 3.30GHz with 4 GB RAM) and each instance was given a single core. We restricted the computational time per instance to 10 minutes for local search experiments and 24 hours for SMT experiments. All produced solutions were verified using HSEval^3^ and are available online.^4^

### Instances

We evaluated our approach on HSTT benchmark instances which can be found on the repository of the International Timetabling Competition 2011 (ITC 2011).^5^ We used the XHSTT-2014 benchmark set, which contains instances that were carefully selected by the ITC 2011 over the years and are meant to be interesting test beds for researchers. Additionally, we included every instance used in the competition (these two sets of instances overlap). We note that out of 39 instances we can currently model 23 instances with our approach. The remaining instances can not currently be modeled with our bitvector approach. This way we took into consideration all relevant HSTT instances which our approach can solve, to the best of our knowledge.

In the instances, the number of time slots ranges from 25 to 125, number of resources from 8 to 99, number of events from 21 to 809 with total event duration from 75 to 1912. These numbers vary heavily from instance to instance. We do not provide detailed information, but direct the interested reader to Post et al. (2012, (2014).

### Bitvectors and local search

We have implemented basic variants of hill climbing and simulated annealing local search solvers for XHSTT using the presented bitvector approach to model XHSTT and calculate constraint violations. For comparison purposes, we have implemented the exact same algorithms using the engine KHE for calculating the constraint costs.

#### Brief discussion on the implementation

In KHE the solution consists of a number of subevents and their assigned times. It is important to note that subevents of the same event are allowed to clash with each other (constraints like Avoid Clash Constraints will penalize such solutions). We now discuss this particular situation in more detail, first by giving an example in KHE and then viewing the same situation with our model.

In KHE, for example, a math lesson of duration four hours can be split to two subevents with duration of two hours. If the first and second subevents are scheduled to take place at $Monday\ 9\ am$ and $Monday\ 10\ am$ (respectively), we will notice that there is an overlap at $Monday\ 10\ am$, because the second subevents starts while the first subevent is still taking place. Therefore, we have a clash of subevents. This is treated as any other clash and the appropriate constraints such as avoid clash constraints apply.

However, in our general bitvector model we cannot have this situation as clashing subevents of the same event is not possible. Instead, for the previous example, the exact same solution using our model could be modeled such that one subevent of duration two starts at $Monday\ 9\ am$, another subevent of duration one starts at $Monday\ 11\ am$, and the event would have one hour of lessons unassigned. In this scenario assign time constraints would penalize such an assignment rather than avoid clash constraints as in KHE.

For the local search implementation we modified our model to take into account subevents. This is done by assigning a bitvector to each subevent. The number of subevents for each event is obtained after generating an initial solution. This modification introduces difficulties when checking some constraints, as in some cases one needs to check for an event whether it has multiple subevents starting at the same time, but this is done to make our implementation more similar to KHE.

We note that we believe the way our general model treats clashing subevents is more natural and appropriate, apart from it being simpler to calculate for our model when compared to the modification described above. For example, we find it unintuitive to allow a lesson to take place in the same time more than once, and that one can avoid violating assign time constraints by creating a new subevent and assigning it a time in which another subevent of the same event is taking place, thus shifting the violation towards Avoid Clashes Constraint. A possible approach would be to encode subevents as separate events and modify the appropriate constraints to accommodate for this (e.g. Spread Events Constraint), but this would not eliminate our first concern. However, we agree that this is somewhat debatable and do not pursue further discussion on this in the following text.

#### Comparison of KHE and bitvectors

KHE is the leading open source software library for the general high school timetabling. It offers users a lot of useful functionality when implementing XHSTT algorithms and has its own solvers as well.

The reason we chose simulated annealing and hill climbing is because they are closely related techniques to GOAL [the winner of the ITC 2011 Brito et al. (2012)], as well as the improvements made later on Fonseca et al. (2015), Fonseca and Santos (2014). GOAL has been implemented using KHE, which is why we chose to compare our approach with KHE. We also use KHE to generate an initial solution.

Events are split into one or more subevents. Regarding the local search algorithms, two local search moves are considered: moving a randomly selected subevent to a new random time and swapping the assigned times of two randomly selected subevents. These moves are chosen because they have been used in Brito et al. (2012) and Fonseca and Santos (2014). The algorithm by itself is a simplified version of the mentioned state-of-the-art algorithms. We deliberately keep the algorithm as simple as possible because the aim is to compare our modeling approach with KHE regarding the number of iterations.The algorithm implemented is described in Algorithm 1, which is a basic simulated annealing algorithm (one gets a variant of hill climbing by omitting the second *or* part of the outer *if* statement).
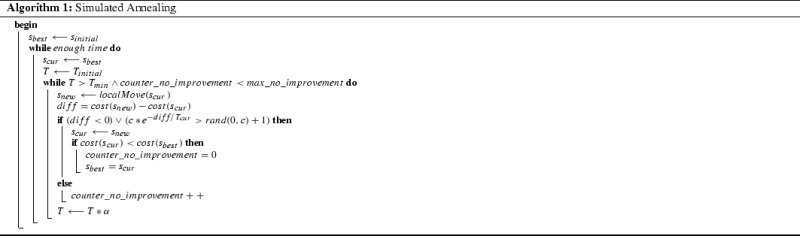


In experiments, the following parameters were used: $T_{initial} = 0.1$, $T_{min} = 0.01$, $\alpha = 0.99$, $max\_no\_improvement = 10000$, $c = 10000$. The cost difference *diff* was calculated as follows (taken from GOAL):

40 $$\begin{aligned} \begin{aligned} diff = (hardCost(s_{new}) - hardCost(s_{cur})) * 10000.0\ \\ +\ \frac{(softCost(s_{new}) - softCost(s_{cur}))}{(hardCost(s_{best}) * 10000.0 + softCost(s_{best}))} \end{aligned} \end{aligned}$$

As a measure for comparison between KHE and our bitvector approach, we compare how many algorithm iterations could be performed in 10 minutes. In Table 1, we present both the objective value and number of iterations performed. The instances in the upper part of the table (separated by the bold horizontal line) represent instances that were used in the final phase of ITC 2011, while the other instances were used in previous phases. We note that the running times for simulated annealing and hill climbing were very similar, therefore we only present one table.

**Table 1:**
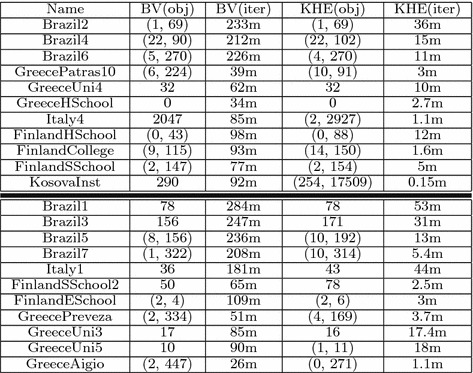
Comparison of the bitvector approach and KHE for basic simulated annealing and hill climbing

In each example our implementation managed to produce more iterations, with the results being mostly better. In some cases less iterations turned out better because of the stochasticity of the algorithms used. We excluded the instance NetherGEPRO because the generation of initial solution took more than the allowed computational time.

We used simplified variants of hill climbling and simulated annealing, because we wanted to show that the bitvector implementation can be used effectively in local search techniques and that it is possible to model the whole problem with the bitvector approach. As we experiment with very simple local search techniques the results are not competitive, but we can see that in each example our implementations produces more iterations.

We believe the improvements come from the data structures used, as they are very compact and simply consist of bitvectors. This makes certain constraints easy to calculate, but more importantly for simulated annealing it allows the solver to efficiently restart from another solution by copying the bitvector data structure which can be done very fast.

When calculating the cost function after performing a local move, KHE and our approach both recalculate costs for the affected resources and events, but the main difference is that KHE recalculates only part of the constraint, while we calculate the complete constraint cost. In our bitvector implementation, in some cases considering only a part of the constraint would not make a difference (e.g. avoid unavailable times constraint), but other constraints might benefit from it, although this has so far not been explored.

Although our implementation for simulated annealing and hill climbing as a whole currently shows better results than when using KHE, we cannot make a general claim that our modeling is better than the approach used by KHE. Indeed, it could be that KHE is more efficient in particular solution components, but this is hard to evaluate as it is difficult to view algorithm components isolated. Nevertheless, our results show that our modeling approach is a useful modeling approach for XHSTT and can be used as it is by local search techniques and SMT.

### Bitvectors for SMT

We evaluated modeling HSTT with bitvectors for Satisfiability Modulo Theories (SMT). The developed bitvector modeling is suitable to be used for solving XHSTT with SMT solvers which provide tools for reasoning over bitvectors. To test our approach we used the instances described in Sect. 5.1.

We experimented with the SMT solver Z3 (v4.4.2) (De Moura and Bjørner 2008) with optimization support (Bjørner and Phan 2014) using the *wmax* optimization engine. We chose this solver because, to the best of our knowledge, it is the only active solver that supports optimization over bitvectors. When modeling we used the encoding for cardinality constraints as described in Sect. 4.2 rather than population count instructions (mentioned in 4.2). The reason for doing so was because there is no support for cardinality constraints in the solver.

We restricted the computational time to 24 h with one core. The time to convert an instance from XHSTT to a SMT instance is negligible when compared to the SMT solution process.

**Table 2:**
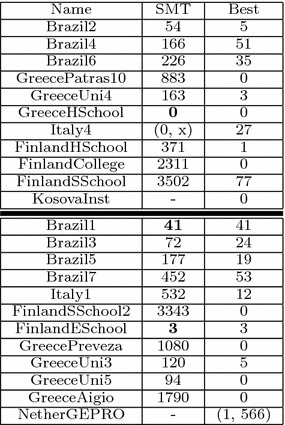
Comparison of SMT and best known results

The comparison of SMT solutions and best known results can be found in Table 2. For each instance we display only the soft constraint cost if the hard constraint cost part is zero. Otherwise, we use a dash to indicate that no feasible solution has been calculated. Our model differentiates only between feasible or not feasible (hard constraints equal to zero or not), that is, it does not give the hard costs. For *ItalyInstance*4, (0, *x*) means that an initial solution was computed but no optimization could be performed. The instances in the upper part of the table (separated by the bold horizontal line) represent instances that were used in the final phase of ITC 2011, while the other instances were used in previous phases. The table only displays instances which we could model with our approach.

In all of the instances (except KosovaInstance and NetherGEPRO), the SMT solver managed to compute an initial solution within a few minutes and do some optimization. For three instances (Brazil1, GreeceHighSchool, and FinlandESchool) optimal solutions were found. However, overall when compared to the best existing results, the SMT method is not competitive, although one must consider that the best known results were obtained without any time or resource limitations.

Therefore, given the current state, it would be best to use our approach to generate an initial solution for a local search, as local search algorithms can struggle in some cases to find a feasible solution [e.g. see Table 2 in Fonseca and Santos (2014)]. Finally, SMT solvers are continuously being improved and future developments of SMT optimization will directly improve our results.

## Conclusion

In this paper, we have shown that the general HSTT problem (Post et al. 2014) (XHSTT) can indeed be encoded using theory of bitvectors, despite the generality of the specification. We presented a complete and detailed modeling in the general sense as required by the specification under the assumption that resources have been preassigned to events, but also have sketched how the model can be extended and discussed some important special cases.

To show the usefulness of our modeling, the bitvector encoding has been applied for calculation of constraint deviations in local search algorithms such as hill climbing and simulated annealing. Our approach is compared to the leading and efficient engine KHE, which has been used to determine constraint violations in state-of-the-art approaches for XHSTT. The experimental results indicate that our implementation of the bitvector modeling is useful and can be used for local search algorithms for HSTT.

Additionally, our model is used to encode XHSTT as a Satisfiability Module Theory (SMT) problem. Our SMT approach managed to find feasible solutions and perform some optimization (in three cases, optimal results were computed). Although the current SMT approach can not outperform the state-of-the-art solvers for XHSTT, the generality of modeling is beneficial, because SMT solvers are continuously improved and in the future they could be used to solve more efficiently XHSST problems based on our encodings. Furthermore, XHSST problems are very interesting benchmarks for the evaluation of SMT solvers and we plan to submit our encodings to SMT-LIB that is an international initiative aimed at facilitating research and development in Satisfiability Modulo Theories (SMT). Apart from that, with our approach we can compute initial solutions which can be used in local search algorithms, since such algorithms can struggle in some cases to find a feasible solution.

For the future work, we plan to investigate the use of bitvector approach for other heuristic techniques. Furthermore, it would be interesting to consider the development of a large neighborhood search algorithm that will utilize SMT solving.

## Footnotes

1
http://www.utwente.nl/ctit/hstt/itc2011/welcome/.

2 As defined by Eq. (34).

3
http://sydney.edu.au/engineering/it/~jeff/hseval.cgi.

4
http://www.dbai.tuwien.ac.at/user/demir/XHSTT_SMT.tar.gz.

5
http://www.utwente.nl/ctit/hstt/itc2011/welcome/.
